# Educational level as a predictor of the incidences of non-communicable diseases among middle-aged Japanese: a hazards-model analysis

**DOI:** 10.1186/s12889-019-7182-6

**Published:** 2019-07-01

**Authors:** Takashi Oshio, Mari Kan

**Affiliations:** 1 Institute of Economic Research, Hitotsubashi University, 2-1 Naka, Kunitachi, Tokyo, 186-8603 Japan; 2 School of Economics, University of Hyogo, 8-2-1 Gakuen-Nishi-machi, Nishi-ku, Kobe, Hyogo 651-2197 Japan

**Keywords:** Educational level, Hazards model, Relative index of inequality, Non-communicable diseases

## Abstract

**Background:**

It is well known that there are educational inequalities in incidences of non-communicable diseases (NCDs). Unlike most preceding studies, this study examined this issue using a hazards model analysis, with specific reference to the potential mediating effects of socioeconomic status (SES), other than educational level, and health behaviour as well as gender differences.

**Methods:**

Data were obtained from a 12-wave longitudinal nationwide survey conducted from 2005 to 2016 with middle-aged individuals in Japan. Participants included 31,210 individuals (15,127 men and 16,083 women) who were aged 50–59 years at wave 1. Incidences of six NCDs (diabetes, heart disease, stroke, hypertension, hyperlipidaemia, and cancer), initially diagnosed between waves 2 and 12, were considered. Cox proportional hazards models were estimated to examine their associations with educational level, adjusted for baseline SES and health behaviour. Educational inequalities were measured by the relative indices of inequality (RII).

**Results:**

Lower educational level was associated with higher incidences of diabetes and stroke among both men and women, and with hypertension only among women. After controlling for baseline SES, health behaviour, and regional areas, the RII ranged from 1.37 (95% confidence interval [CI]: 1.02–1.85) for stroke among men to 2.65 (95% CI, 2.09–3.36) for diabetes among women. Small to moderate parts (0.0–32.7%) of the RII were explained by baseline SES and health behaviour. A negative association with education was observed for diabetes and hypertension among women.

**Conclusions:**

Results underscored the importance of educational level as a predictor of the incidences of selected NCDs, especially among women, with limited mediating effects of other SES and health behaviour.

## Background

It is well known that health outcomes are closely related to educational level [1]. Specifically, lower levels of education have been found to be related to higher incidence and prevalence of non-communicable diseases (NCDs) [2, 3]—including cardiovascular and cerebrovascular diseases [4–7], cancers [8, 9], diabetes [10–12], hypertension [13], and chronic respiratory diseases [14]—in addition to higher levels of cause-specific and all-cause mortality [15–17]. However, most of the preceding studies on educational inequalities in NCD incidence have been prospective cohort analyses, which focused on the prevalence or cumulative incidence over the follow-up period among respondents with no baseline disease (e.g. [5, 11, 14]), or (repeated) cross-sectional analyses, which compared the prevalence of the diseases among respondents with different levels of educational attainment (e.g. [2, 4, 6, 18]). In comparison, hazards model analyses, which aimed to relate the time passage before the incidence of the disease to educational level, have been relatively scarce [12].

A related issue that should be further addressed is to what extent educational inequalities in NCD incidence are mediated by socioeconomic status (SES), other than educational level, and health behaviour. Low income and unstable job statuses, which are likely linked to low educational level, are reasonably predicted to raise the risk of NCD incidence, as documented by previous studies [2, 3]. Similarly, some types of health behaviours, such as smoking, heavy alcohol drinking, physical inactivity, and unhealthy diet, are expected to mediate the impact of educational level on NCD incidence [1]. To be sure, many studies have assessed the importance of these factors in explaining the educational inequalities in mortality [19–22], worsening frailty [23], and mental health [24]. However, the meditating effects of these factors on educational inequalities in NCD incidence have been largely understudied. If the mediating effects are limited, policy interventions to improve SES or promote healthy lifestyle after the completion of education would not be expected to be effective in moderating educational inequalities in health during later life. Indeed, some studies have provided evidence for the limited mediating effects of SES and health behaviour on worsening frailty [23] and NCD prevalence [18].

It is also interesting to explore gender differences in the association between educational inequalities and NCD incidence. Some studies have found steeper educational gradients in the incidence of some types of NCD among women than among men [2, 5]. Gender differences may also depend on the type of NCD and there may be also gender differences in contributions of SES and health behaviour in explaining educational inequalities [25].

Considering the obtained knowledge and limitations of the existing literature, the current study conducted a hazards model analysis to examine educational inequalities in NCD incidence, using the 12-wave longitudinal data obtained from nationwide population-based surveys in Japan. The hazards models were estimated for six types of NCDs, and educational inequalities were compared across them. The results are expected to help evaluate the relevance of the observations obtained by preceding prospective cohort and cross-sectional studies. In addition, the extent to which education inequalities in NCD incidence were mediated by SES, other than educational level, and health behaviour was examined. Specifically, household-size adjusted income and job status as well as four types of health behaviour (smoking, heavy alcohol drinking, physical inactivity, and unhealthy diet) at baseline were examined as potential mediators. Furthermore, gender differences in education inequalities were explored.

Japan faces a growing burden of NCDs, while its rate of longevity is one of the highest in the world; NCDs accounted for 54.5% of total causes of death in 2017, and deaths caused by cancers and heart diseases, the top two causes of death among NCDs, increased from 11.6 and 8.7 per million, respectively, in 1970 to 29.9 and 16.4 per million in 2017 [26]. The increasing burden of NCDs has enhanced the need for health promotion policies to prevent and control NCDs, especially under extended longevity [27, 28]. The current study focused on the risks of NCDs among middle-aged Japanese, who were 50–59 years old at baseline, given that the prevalence of key NCDs has been shown to increase substantially during the middle age years [26].

## Methods

### Study sample

Data were obtained from a nationwide, 12-wave panel survey, the Longitudinal Survey of Middle-Aged and Older Adults, conducted by the Japanese Ministry of Health, Labour and Welfare (MHLW) [29] each year from 2005 to 2016. Samples in wave 1 were collected nationwide from individuals between the ages of 50 and 59 years in November 2005, through a two-stage random-sampling procedure. First, 2515 districts were randomly selected from 5280 districts used in the MHLW’s nationwide, population-based Comprehensive Survey of the Living Conditions, conducted in 2004. The 5280 districts, in turn, were randomly selected from approximately 940,000 national census districts. Second, depending on the population size of each district, 40,877 residents, aged 50–59 years as of 30 October 2005, were randomly selected.

The questionnaires were manually delivered to the participants’ homes and they were asked to complete them by November 2; the completed questionnaires were collected several days later. A total of 34,240 individuals responded (response rate: 83.8%). Waves 2–12 of the survey were conducted from 2006 to 2016. The questionnaires of the subsequent waves were only mailed to those participants who had mailed back the completed questionnaires from the previous wave or the one prior to that (average attrition rate of 4.0% in each wave). No new respondents were added after wave 1. After removing the respondents missing key variables from the statistical analysis, the responses of 31,210 individuals (15,127 men and 16,083 women) were analysed. To capture the educational inequalities in each NCD incidence, the sample used in statistical analysis was further limited to those who did not report the incidence of each disease at the baseline (wave 1). Thus, the number of respondents used for the statistical analysis ranged from 25,867 to 30,824, depending on the diseases. The data from the 2005–2014 surveys were used in the corresponding author’ previous study [29], and the newly released data from the 2015 and 2016 surveys were additionally used in this study.

### Measures

#### NCDs

Six types of NCDs—diabetes, heart disease, stroke, hypertension, hyperlipidaemia, and cancer—were considered. For each NCD, the respondents were asked whether they had been diagnosed with the NCD by a medical doctor at the time of the survey [30]. Although a few examples were presented for some NCDs to the respondents in the survey (e.g. angina and cardiac infarction for heart disease), the experience of diagnosis was self-reported. A binary variable, in which a score of 1 was allocated to the respondents who reported being diagnosed and 0 to others, was constructed.

#### Educational level

The survey asked respondents to choose their final educational attainment from among (i) junior high school, (ii) high school, (iii) vocational school, (iv) junior college or technical college, (v) college, (vi) graduate school, and (vii) other. These seven categories were condensed into three levels: ‘low’ (i), ‘middle’ (ii, iii, iv, and vii) and ‘high’ (v and vi), which is standardized categorisation of educational attainment in Japan (e.g. [31–33]). In addition, the ridit score of educational level was derived for each educational level by calculating the mean proportion of the population after ranking it from the highest level to the lowest [34, 35]. For instance, if the respondents with high, middle, and low educational levels comprise 30, 50, 20%, respectively, of the sample, the ridit-scores for each educational level are calculated as 0.15 (0.3/2) (high), 0.55 (0.3 + 0.5/2) (middle), and 0.9 (0.3 + 0.5 + 0.2/2) (low), respectively, using midpoints for the proportion of the respondents in each level. A higher ridit score corresponds to a lower educational level. This score was used as a continuous variable in the regression analysis.

#### Baseline SES

Regarding baseline SES other than educational attainment, household income and job status were considered. As for household income, reported household spending was used as its proxy for three reasons: dependent wives tended to report no income, household spending was expected to represent their standard of living more accurately, and limiting to respondents reporting income reduced the sample size substantially. Household spending was household-size adjusted, by dividing the reported amount by the square root of the number of household members. Then, a binary variable of ‘low income’ was constructed by allocating a score of 1 to the respondents whose (household size adjusted) household spending belonged to the lowest quartile and 0 to others.

Regarding job status, the respondents were first asked whether they had a paid job, and if they responded ‘yes’, they were asked to choose their job status from among (i) self-employed, (ii) family worker, (iii) executive of a corporation/organisation, (iv) regular employee, (v) part-time employee, (vi) dispatched employee, (vii) temporary employee, and (viii) engaged in piecework at home, and (ix) other. In this study, the job status was categorised into ‘no job’, ‘stable job’ (i, iii, iv), and ‘unstable’ job (ii, v–ix), and ‘stable job’ was used as a reference category.

#### Baseline health behaviour

Four types of health behaviours—smoking, heavy alcohol drinking, physical inactivity, and unhealthy diet—were considered and each of them was expressed as a binary variable. Respondents who answered ‘yes’ to the question ‘Do you smoke currently?’ were considered current smokers. Heavy alcohol drinking for men was defined as an intake of more than three *go* (540 ml) of Japanese sake or an equivalent amount of alcohol every day, which corresponds to about 60 g of pure alcohol. This threshold was halved for women. These definitions were based on a study that showed that maintaining alcohol consumption below 46 g/day (for men) and 23 g/day (for women) appeared to minimise the risks of mortality in a Japanese population [36]. Physical inactivity was defined as engaging in no leisure-time physical activity. Finally, regarding diet, respondents were asked whether they were making an effort on a daily basis to (i) pay attention to the amount of a meal, (ii) eat variety of foods as part of a balanced diet, or (iii) take vitamins or minerals in tablet, capsule, powdered or drinkable form. Unhealthy diet was defined as making none of these efforts.

#### Covariates

Age, self-rated health (SRH), and regional areas at the baseline were used as covariates. For age, binary variables for each age were constructed. For SRH, respondents were asked to choose from among ‘very good’, ‘somewhat good’, ‘somewhat poor’, ‘poor’, and ‘very poor’ as a response to the question ‘What is the current condition of your health?” Binary variables for each response were constructed. Regional areas were included as covariates to account for the potential regional heterogeneity of NCDs and other variables. Binary variables for each of eight areas (Hokkaido, Tohoku, Kanto, Chubu, Kinki, Chugoku, Shikoku, and Kyushu) were used in regression models.

### Statistical analysis

As descriptive analysis, trends in the numbers of respondents who experienced diagnosis of each NCD at any time between waves 2 and 12 across three educational levels were examined. The statistical significance of this trend was evaluated by ‘*p* for trend’, based on a chi-square statistic of the trend. In what follows, the trend of health on education—that is, the extent to which the health outcome changes in response to an increase in educational level—is referred to as educational ‘gradient’ in health.

Following the analysis of variance (ANOVA) to examine the heterogeneity of the prevalence of each NCD across regional areas at baseline, the regression analysis began with examining the association between the ridit score of educational level and each variable of baseline SES and health behaviour—which are considered potential mediators of the impact of educational level in NCD incidence—by logistic regression models, using baseline age and SRH as covariates. The relative index of inequality (RII) by educational level in each outcome was obtained by taking the exponent of the estimated coefficient of the (continuous) ridit score of education in the logistic regression model, in the same way as calculating the odds ratio (OR) of a binary variable [37, 38]. Thus, the RII indicates the ratio of the odds of each outcome at the lowest educational level (with the ridit score = 1) to those at the highest level (with the ridit score = 0). The RII has been often used to assess educational inequalities in health outcomes by preceding studies [4, 18, 24, 39].

Then, two types of Cox proportional hazards model (Models 1 and 2) were estimated to examine the association between educational level and each NCD incidence. Model 1 explained each NCD incidence solely by the ridit score of educational level, while Model 2 added baseline SES and health behaviour as explanatory variables to Model 1. Both models included baseline ages and SRH as covariates. As with the case of logistic model, the estimated coefficient, if transformed to the hazard ratio (HR), indicates the RII by educational level in NCD incidence. Moreover, the extent of attenuation in the estimated HR from Model 1 to Model 2 indicates how baseline SES and health behaviour jointly mediated the educational inequalities in NCD incidence.

Finally, gender differences in educational inequalities were examined by adding the binary variable for females and its interaction term with the ridit score to Model 2 and using the entire sample. The statistical significance of the estimated HR of the interaction term was used to assess the gender differences.

The software package Stata (Release 15) was used for the statistical analysis [40].

## Results

The key features of the study sample are summarised in Table 1, along with the gender difference and its statistical difference for each variable. Low, middle, and high educational levels comprised 19.4, 55.2, and 25.4%, respectively, for men, and 18.4, 74.9, and 6.7%, respectively, for women, indicating higher educational attainment among men compared to women. The proportion of stable job status was much higher among men (84.2%) than among women (25.4%). Women displayed healthier lifestyle than men in all four aspects (smoking, heavy alcohol drinking, physical inactivity, and unhealthy diet).

**Table 1:** Key features of the study sample at baseline

		All		Men		Women		Difference	
								(Men –Women)	
		*N*	(%)	*N*	(%)	*N*	(%)	(% point)	*p*-value
Educational attainment									
Low	Junior high school	5902	(18.9)	2937	(19.4)	2965	(18.4)	1.0	0.027
Middle	High school	17,940	(57.5)	7868	(52.0)	10,072	(62.6)	−10.6	< 0.001
	Junior college	2248	(7.2)	368	(2.4)	1880	(11.7)	−9.3	< 0.001
	Other	205	(0.7)	116	(0.8)	89	(0.6)	0.2	0.020
	Total	20,393	(65.3)	8352	(55.2)	12,041	(74.9)	−19.7	< 0.001
High	College	4658	(14.9)	3617	(23.9)	1041	(6.5)	17.4	< 0.001
	Graduate school	257	(0.8)	221	(1.5)	36	(0.2)	1.2	< 0.001
	Total	4915	(15.7)	3838	(25.4)	1077	(6.7)	18.7	< 0.001
Socioeconomic status									
Low income		6788	(21.7)	3077	(20.3)	3711	(23.1)	−2.7	< 0.001
Job status									
Stable job		16,829	(53.9)	12,738	(84.2)	4091	(25.4)	58.8	< 0.001
Unstable job		8364	(26.8)	1380	(9.1)	6984	(43.4)	−34.3	< 0.001
No job		6017	(19.3)	1009	(6.7)	5008	(31.1)	−24.5	< 0.001
Health behavior									
Smoking		9346	(29.9)	7307	(48.3)	2039	(12.7)	35.6	< 0.001
Heavy drinking		1420	(4.5)	1293	(8.5)	127	(0.8)	7.8	< 0.001
Physical inactivity		15,348	(49.2)	8141	(53.8)	7207	(44.8)	9.0	< 0.001
Unhealthy diet		7444	(23.9)	4597	(30.4)	2847	(17.7)	12.7	< 0.001
Self-rated health									
Very good		2465	(7.9)	1240	(8.2)	1225	(7.6)	0.6	0.057
Good		9747	(31.2)	4778	(31.6)	4969	(30.9)	0.7	0.189
Somewhat good		13,078	(41.9)	6145	(40.6)	6933	(43.1)	−2.5	< 0.001
Somewhat poor		4322	(13.8)	2172	(14.4)	2150	(13.4)	1.0	0.011
Poor		1080	(3.5)	532	(3.5)	548	(3.4)	0.1	0.597
Very poor		277	(0.9)	151	(1.0)	126	(0.8)	0.2	0.043
Age	*M*	54.7		54.7		54.7		0.0	0.460
	*SD*	(2.7)		(2.7)		(2.7)			
Household spending^a^	*M*	188.9		195.0		183.1		11.9	< 0.001
(monthly, thousand yen)	*SD*	(180.3)		(206.4)		(151.1)			
*N*		31,210		15,127		16,083			

^a^Household-size adjusted

The prevalence of each NCD over the sample period was compared by educational level in Table 2. Without controlling for any other factor, an inverse educational gradient was observed for diabetes and stroke among both genders, while such a gradient was observed for heart disease and hypertension only among women. Hyperlipidaemia was positively associated with educational level among both men and women, and cancer was not related to it among either men or women.

**Table 2:** Prevalence (%) of each non-communicable disease over 11-wave, follow-up by educational level

Educational level	All	Low	Middle	High	*p* for trend	*N*
Men (*N* = 15,127)						
Diabetes	13.7	15.4	14.2	11.4	< 0.001	13,719
Heart disease	9.7	9.8	9.7	9.5	0.687	14,581
Stroke	4.9	5.9	5.0	3.9	< 0.001	14,886
Hypertension	32.9	32.7	33.2	32.5	0.820	12,281
Hyperlipidaemia	23.8	18.0	24.0	28.0	< 0.001	13,792
Cancer	8.6	9.1	8.6	8.3	0.237	14,931
Women (*N* = 16,083)						
Diabetes	8.8	13.4	8.0	5.3	< 0.001	15,333
Heart disease	6.0	7.1	5.8	5.8	< 0.001	15,803
Stroke	3.2	4.3	3.0	1.7	< 0.001	15,938
Hypertension	23.8	27.7	23.4	18.6	< 0.001	13,586
Hyperlipidaemia	25.7	20.7	26.8	28.3	< 0.001	14,706
Cancer	7.5	7.7	7.4	7.1	0.528	15,762

The results of the analysis of variance, which tested the null hypothesis that there was no difference in prevalence of each NCD across regional areas, are presented in Table 3. Judging by the *p*-values, six out of twelve gender-NCD combinations revealed regional heterogeneity. This result indicated the need to control for regional areas in regression analysis.

**Table 3:** Results of the analysis of variance to test the equality of prevalence across eight regional areas^a^ at baseline for each non-communicable disease

	Men			Women		
	*F*-statistics	*p*-value	*N*	*F*-statistics	*p*-value	*N*
Diabetes	1.21	0.293	13,719	2.64	0.010	15,333
Heart disease	1.45	0.181	14,581	0.49	0.845	15,803
Stroke	3.31	0.002	14,886	2.03	0.048	15,938
Hypertension	1.74	0.096	12,281	1.16	0.320	13,586
Hyperlipidaemia	1.56	0.141	13,792	2.10	0.041	14,706
Cancer	1.70	0.105	14,931	2.47	0.016	15,762

^a^Hokkaido, Tohoku, Kanto, Chubu, Kinki, Chugoku, Shikoku, and Kyushu

The estimated RIIs of educational level for baseline SES and health behaviour are summarised in Table 4. To assess the RII of educational level, the estimated OR of each outcome responding to an increase in the ridit score of educational level from 0 (highest) to 1 (lowest) was calculated. Judging by the estimated ORs, lower educational levels were positively associated with low income and unstable job status. The association between lower educational levels and no job was positive for men but negative for women; the latter result probably reflected the existence of highly-educated, dependent wives with no paid job. All four types of unhealthy behaviour were also found to be positively associated with lower educational level; these educational gradients were consistent with the argument that lower SES and unhealthy behaviour can explain educational inequalities in NCD incidence.

**Table 4:** Estimated associations of educational level with baseline socioeconomic status and health behaviour^a^

	Men (*N* = 15,127)			Women (*N* = 16,083)	
	OR^b^ (RII)		95% CI^c^	OR (RII)	95% CI
Socioeconomic status					
Low income	3.73***		(3.14, 4.42)	3.25***	(2.72, 3.87)
Job status					
Unstable job	2.64***		(2.10, 3.33)	1.98***	(1.70, 2.30)
No job	2.42***		(1.83, 3.20)	0.82*	(0.70, 0.97)
Health behaviours					
Smoking	2.80***		(2.45, 3.21)	2.99***	(2.40, 3.73)
Heavy drinking	1.26		(0.99, 1.60)	2.79*	(1.24, 6.29)
Physical inactivity	4.06***		(3.54, 4.66)	4.18***	(3.57, 4.88)
Unhealthy diet	1.47***		(1.28, 1.70)	2.29***	(1.89, 2.77)

^***^ *p* < 0.001, ^**^ *p* < 0.01, ^*^ *p* < 0.05

^a^Adjusted for ages and regional areas at baseline

^b^Odds ratio. It indicates the relative index of inequality (RII) of educational level

^c^Confidence interval

**Fig. 1:**
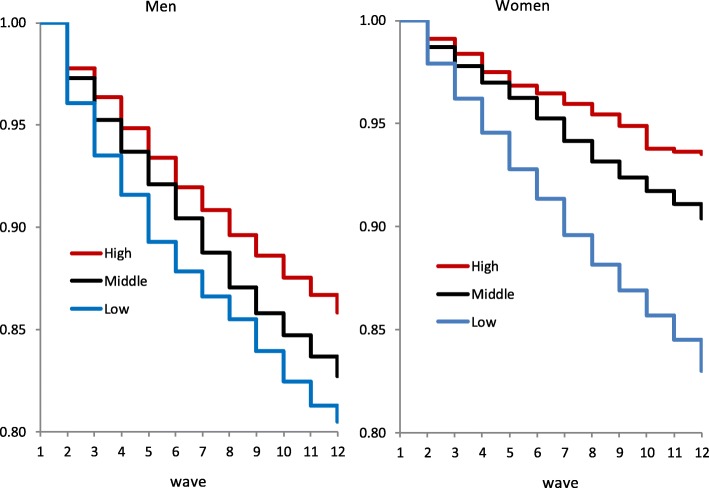
Kaplan-Meier survival estimates by educational level: the case of diabetes

Hazards model analysis began by graphically illustrating the Kaplan-Meier survival estimates by educational level in the case of diabetes (without controlling for any other variable). As shown in Fig. 1, the differences in cumulative incidences of diabetes tended to widen as time passed particularly among women. Educational inequalities in the incidence of diabetes were confirmed more formally by Cox proportional hazards models. The estimation results of Models 1 and 2 for diabetes are presented in Table 5. The HRs of the incidence of diabetes in response to an increase in the ridit score of educational level from 0 (highest) to 1 (lowest) (in both Models 1 and 2) as well as to lower SES and unhealthy behaviour (in Model 2) were calculated. The HR for the ridit score indicates the RII by educational level. The estimated HRs were well above one in all model specifications, confirming the inverse educational gradient in the incidence of diabetes, and the gradient was steeper for women than for men.

**Table 5:** Estimated hazard ratios of diabetes for educational level and baseline socioeconomic status and healthy behaviour

	Men (*N* = 13,719)				Women (*N* = 15,332)			
	Model 1^a^		Model 2^b^		Model 1		Model 2	
	HR^c^	95% CI^d^	HR	95% CI	HR	95% CI	HR	95% CI
Ridit score of educational level	1.49 ***	(1.25, 1.79)	1.41***	(1.18, 1.70)	2.65***	(2.09, 3.36)	2.56***	(2.01, 3.26)
Socioeconomic status								
Low income			0.96	(0.85, 1.07)			0.97	(0.85, 1.10)
Job status								
Unstable job			1.00	(0.86, 1.18)			1.12	(0.98, 1.29)
No job			1.09	(0.91, 1.31)			1.17*	(1.01, 1.36)
Health behaviour								
Smoking			1.14**	(1.04, 1.25)			1.07	(0.91, 1.26)
Heavy drinking			1.23**	(1.06, 1.44)			1.08	(0.59, 1.96)
Physical inactivity			1.10*	(1.00, 1.20)			1.05	(0.94, 1.17)
Unhealthy diet			0.91	(0.83, 1.01)			1.07	(0.94, 1.23)

^***^ *p* < 0.001, ^**^ *p* < 0.01, ^*^ *p* < 0.05

^a^Adjusted for ages, self-rated health, and regional areas at baseline

^b^Adjusted for socioeconomic status and health behaviour as well as ages, self-rated health, and regional areas at baseline

^c^Hazard ratio. Its value of the ridit score of educational level indicates the relative index of inequality (RII) of educational level

^d^Confidence interval

It was also found that among both men and women, the HRs for the ridit score of educational level was not much attenuated and remained highly significant in Model 2, which controlled for baseline SES and health behaviour. Meanwhile, the results of SES and health behaviour in Model 2 were mixed; the incidence was positively associated with smoking, heavy alcohol drinking, and physical inactivity among men and only with no job among women, while no association was observed for any other variable.

Similar Cox proportional hazards models were estimated for other NCDs as well, and their estimated HRs for educational level are summarised in Table 6. Even after controlling for baseline SES and health behaviour, educational inequalities were observed for diabetes and stroke among both men and women and for hypertension only among women. Heart disease and cancer were not associated with educational level, while hyperlipidaemia had a positive association. Only small to moderate parts of the HR were attenuated by baseline SES and health behaviour for NCDs that had inverse educational gradients. When the degree of attenuation was calculated by (HR in Model 1 – HR in Model 2) / (HR in Model 1–1) [22], it was in the range between 0.0% (for hypertension among women) and 32.7% (for stroke among men). It was also observed that attenuation was more limited among women than among men for diabetes and stroke.

**Table 6:** Estimated hazard ratios of each non-communicable disease with respect to educational level

	Model 1^a^		Model 2^b^		Attenuation in HR (%)^e^	*N*
	HR^c^	95% CI^d^	HR	95% CI		
Men						
Diabetes	1.49***	(1.25, 1.79)	1.41***	(1.18, 1.70)	16.3	13,719
Heart disease	0.97	(0.79, 1.19)	0.94	(0.76, 1.16)		14,581
Stroke	1.55**	(1.17, 2.07)	1.37*	(1.02, 1.85)	32.7	14,886
Hypertension	1.00	(0.89, 1.13)	1.04	(0.91, 1.17)		12,281
Hyperlipidaemia	0.55***	(0.48, 0.63)	0.61 ***	(0.53, 0.71)		13,792
Cancer	1.14	(0.92, 1.42)	1.14	(0.92, 1.43)		14,931
Women						
Diabetes	2.65***	(2.09, 3.36)	2.56***	(2.01, 3.26)	5.5	15,333
Heart disease	1.12	(0.83, 1.50)	1.10	(0.82, 1.48)		15,803
Stroke	1.97***	(1.33, 2.91)	1.84**	(1.24, 2.75)	12.4	15,938
Hypertension	1.48***	(1.26, 1.73)	1.48***	(1.26, 1.74)	0.0	13,586
Hyperlipidaemia	0.59***	(0.51, 0.69)	0.64***	(0.55, 0.75)		14,706
Cancer	1.05	(0.80, 1.37)	1.07	(0.81, 1.40)		15,762

^***^ *p* < 0.001, ^**^ *p* < 0.01, ^*^ *p* < 0.05

^a^Adjusted for ages, self-rated health, and regional areas at baseline

^b^Adjusted for socioeconomic status and health behaviour as well as ages, self-rated health, and regional areas at baseline

^c^Hazard ratio. It indicates the relative index of inequality (RII) of educational level

^d^Confidence interval

^e^ (HR in Model 1 – HR in Model 2)/(HR in Model 1–1) × 100%

Another noticeable finding was that, as already observed for diabetes in Fig. 1 and Table 5, the estimated HRs for the ridit score of educational level tended to be higher among women than among men for NCDs whose incidences were negatively associated with educational level. To examine gender differences more formally, Table 7 presents the estimated HRs for the ridit score, women, and their intersection terms, obtained by Model 2 using the entire sample. The estimated HR for the intersection term was significantly higher than one for diabetes and hypertension, indicating sharper inverse educational gradients for these diseases among women than among men. The HR for the intersection term was greater than 1 for heart disease, stroke, and hyperlipidaemia, but non-significant.

**Table 7:** Estimated hazard rates of each non-communicable disease for educational level, using the entire sample^a^

	Ridit score of educational level		Females		Ridit score of educational level × Females		*N*
	HR^b^ (RII)	95% CI^c^	HR	95% CI	HR	95% CI	
Diabetes	1.37***	(1.14, 1.64)	0.41***	(0.34, 0.50)	2.01***	(1.50, 2.69)	29,051
Heart disease	0.91	(0.74, 1.12)	0.58***	(0.47, 0.72)	1.28	(0.90, 1.81)	30,383
Stroke	1.38*	(1.03, 1.84)	0.55***	(0.41, 0.74)	1.35	(0.84, 2.17)	30,823
Hypertension	1.01	(0.90, 1.14)	0.54***	(0.48, 0.61)	1.51***	(1.24, 1.84)	25,866
Hyperlipidaemia	0.60***	(0.53, 0.69)	1.02	(0.91, 1.14)	1.10	(0.90, 1.34)	28,497
Cancer	1.16	(0.93, 1.44)	1.02	(0.83, 1.24)	0.90	(0.64, 1.26)	30,692

^***^ *p* < 0.001, ^**^ *p* < 0.01, ^*^ *p* < 0.05

^a^Adjusted for socioeconomic status, health behaviour as well as ages, self-rated health, and regional areas at baseline

^b^Hazard ratio. Its value of the ridit score of educational level indicates the relative index of inequality (RII) of educational level

^c^Confidence interval

## Discussion

This study examined educational inequalities in NCD incidence using the data obtained from a 12-wave longitudinal nationwide survey of middle-aged individuals in Japan. Unlike most of the preceding studies, this study applied a hazards model analysis to the data over a long follow-up period (11 years) to capture the association between educational level and NCD incidence. In addition, the potential mediating effects of baseline SES and health behaviour as well as gender differences were explicitly considered. Key findings and their implications are summarised as follows.

First, educational inequalities were confirmed for selected types of NCDs. Even after controlling for baseline SES and health behaviour, Cox proportional hazards model regressions showed that lower educational level was positively associated with the incidences of diabetes and stroke among both men and women, and with hypertension only among women. The observations of the inverse educational gradients were largely consistent with those of preceding studies (for diabetes [2, 10–12, 18], stroke [2, 5], and hypertension [2, 13]).

Meanwhile, among both men and women, the incidence of heart disease or cancer had no educational inequalities and the incidence of hyperlipidaemia was positively associated with educational level. Notably, the observed positive association between educational level and hyperlipidaemia was somewhat surprising, but it was consistent with the observation among Korean male adults [41]. One possible reason to explain this result is that higher educational level may lead to a dietary style linked to higher risks of hyperlipidaemia. Indeed, total energy and fat intakes have been found to be positively associated with household expenditure, which is closely linked to educational level, among both male and female adults in Japan [42], possibly because higher-educated, higher-SES individuals have more chances to dine out [41].

Although identifying the reasons to account for the differences across NCDs is beyond the scope of this study, the findings generally underscore the importance of educational level as a key predictor of NCD incidence. To be sure, it cannot be concluded from the results of this study that educational level is a key determinant of NCDs. However, it can be reasonably argued that educational level can be a reliable signal of the risk of the incidences of selected NCDs in later life.

Second, the results revealed that the mediating effects of SES, other than educational attainment, and health behaviour on NCD incidence are relatively limited. Lower income and job status as well as unhealthy behaviour have been found to explain income inequalities in health outcomes [19–22, 24]. Actually, the results showed that lower educational level tended to increase risks of low income, unstable job status, smoking, heavy alcohol drinking, physical inactivity, and unhealthy diet, and that some of these factors modestly raised the risk of NCD incidence. However, inclusion of these factors led to just low-moderate attenuation in educational inequalities of NCD incidence, a result consistent with the observations in some preceding studies [18, 23]. This finding suggests that educational attainment as a predictor of NCD incidence is mostly independent of these factors. However, there can be other potential mediators—such as obesity [19], health literacy [43], social participation [23], and accessibility to health care service—that were not considered in this study. Nevertheless, the results point to limited effectiveness of policy interventions to improve SES or promote healthy lifestyle in reducing the educational inequalities in NCD incidence.

Finally, the results showed significant gender differences in educational inequalities in the incidences of diabetes and hypertension. Similar observations have been obtained in preceding studies for diabetes [2, 18] and hypertension [2]. The exact reason for these differences and also the reason for no gender differences observed for other diseases were not addressed in this study. However, it might be possible that gender differences in social roles, exposures to stressors, and coping strategies may confound gender differences in educational inequalities in NCD incidence [44, 45].

It should be noted that this study had several limitations, besides the observations being limited to middle-aged Japanese people and the reliability of self-reported NCD diagnosis. First, SES and health behaviour of the sample are likely to change across the periods possibly due to health reasons, even though educational attainment can be considered mostly fixed in this study sample. Hence, focusing exclusively on these factors at the baseline may not precisely capture their mediating effects. Second, the results are likely to have been affected by the definitions of health behaviours and other variables. For instance, while physical inactivity was defined as engaging in no leisure-time physical activity in this study, people can be very active in their mainstream life without engaging in leisure-time physical activity. Third, as mentioned above, there are potentially other mediators than those of SES and health behaviour considered in this study for educational inequalities in NCD incidence. Adding these factors in the analysis, the attenuation of estimated educational inequalities from Model 1 to Model 2 may be reduced, possibly affecting the interpretation regarding the degree of independent impact of educational level on NCD incidence. Fourth, respondents who dropped out without incidence were censored in each regression model, as is usually the case of hazards model analysis. Although the average attrition rate was as low as 4% in each wave, neglecting the possibility of the attrition due to the incidence may have led to an underestimation of the risk of incidence.

## Conclusions

This study underscores educational inequalities in the incidences of selected NCDs, especially among women. Inverse educational gradient in NCD incidence was not much attenuated by adjusting for mediating effects of baseline SES and health behaviour. Further analysis is needed to identify the mechanism linking educational level and incidences of each specific NCD. Still, the observation that educational level was associated with NCD incidence in a relatively non-mediated manner has important implications for public health. Educational level can be utilized as a reliable signal of the risk of NCD incidence in general, suggesting that the policy measures to reduce such a risk should be targeted to individuals with lower educational level. It can be also argued that policy support to help socioeconomically disadvantaged children to enhance educational attainment is recommended to reduce inequality in health in later life.

## Data Availability

The data that support the findings of this study are available from the MHLW but restrictions apply to the availability of these data, which were used under licence for the current study, and so are not publicly available. Data are, however, available from the authors upon reasonable request and with permission of the MHLW.

## Abbreviations

HR: Hazard ratio
MHLW: Ministry of Health, Labour and Welfare
NCD: Non-communicable diseases
OR: Odds ratio
RII: Relative index of inequality
SES: Socioeconomic status
SRH: Self-rated health
